# Extraction and antioxidant activity of total triterpenoids in the mycelium of a medicinal fungus, *Sanghuangporus sanghuang*

**DOI:** 10.1038/s41598-019-43886-0

**Published:** 2019-05-15

**Authors:** Chengshan Cai, Jiexin Ma, Chunrui Han, Yi Jin, Guozhu Zhao, Xiangwei He

**Affiliations:** 1 College of Biological Sciences and Biotechnology, Beijing Forestry University, Beijing, 100083 China; 2 College of Materials Science and Technology, Beijing Forestry University, Beijing, 100083 China

**Keywords:** Biocatalysis, Fungal biology

## Abstract

The scientific name of the traditional Chinese medicinal fungus, *Sanghuang*, has been clarified and confirmed that it is a new species -*Sanghuangporus sanghuang* in the recently discovered genus, *Sanghuangporus*. To maximize the yield of the active ingredients such as the triterpenoids from authentic *Sanghuangporus sanghuang*, four parameters of the extraction process, including the extraction time, solid–liquid ratio, extraction temperature, and ethanol concentration were determined. The Box–Behnken experimental design and the response surface method were used to optimize the triterpenoid extraction processes of *Sanghuangporus sanghuang* mycelium. The results showed that the parameters of the triterpenoid extraction processes were not simple linear relationships. Optimum conditions of ultrasonic extraction required an 80% ethanol concentration, a 1:20 solid–liquid ratio, a 20-min extraction time, and a 60 °C extraction temperature, to obtain a maximum triterpenoid extraction of 13.30 mg/g. Antioxidant capacity tests showed that the *Sanghuangporus sanghuang* triterpenoids had high clearance capabilities for hydroxyl free radicals, superoxide anions, 2,2-diphenyl-1-picrylhydrazyl free radicals, and 2,2’-azinobis-(3-ethylbenzthiazoline-6-sulfonate) radicals, indicating that the *Sanghuangporus sanghuang* triterpenoids had high antioxidant activities.

## Introduction

The first use of the medicinal fungus *Sanghuang* can be traced back to 2,000 years ago in China. According to *The Theory of Medicinal*, *Sanghuangporus sanghuang* tastes bitter and is used as a traditional Chinese medicine for the treatment of diarrhea, night sweats, metrorrhagia, drench, stomach pain, prolapse of spilled blood, leucorrhea, and amenorrhoea. *Shennong’s Herbal Classic of Materia Medica* stated that long-term use of *Sanghuangporus sanghuang* can prolong life, detoxify, and improve digestion^1^. In 1968, Ikekawa *et al*. found that the sarcoma cell line S-180 was inhibited by 96.7% in mice when treated with a water extract of the *Sanghuang* fruiting body^2^. The medicinal functions of *Sanghuang* have since been studied by many researchers, who have characterized its antitumor and antioxidant properties. *Sanghuang* is considered one of the most effective anticancer drugs found in higher fungi and has been extensively studied as a medicinal fungus^3,4^. However, the scientific name ‘*Sanghuang’* has been controversial, and it has been mistakenly reported as *Inonotus linteus* (Berk and Curtis) Teixeira, *Phellinus igniarius* (L.) Quél., and *Phellinus baumii* Pilát etc. It has been suggested that the authentic *Sanghuang* should be renamed *Sanghuangporus sanghuang*, as a new species has been discovered that only grows on living mulberry trees^5–9^.

Studies of *Sanghuang* showed that its main components are polysaccharides, flavonoids, and triterpenoids. Triterpenoids are a class of bioactive substances in medicinal fungi, but their antitumor and antioxidant properties have been less studied than those of polysaccharides^10^. They have many functions, such as inhibiting histamine release, lowering blood pressure, and protecting the liver^11^. Based on its “targeted killing,” triterpenoids have recently been studied as possible antitumor agents^12–14^; thus, the extraction process and the antioxidant capacity of triterpenoids in authentic *Sanghuangporus sanghuang* are very important.

Wild *Sanghuang* fruit grows extremely slowly and is difficult to find. Although *Sanghuang* is grown commercially, its authenticity is often debated. Liquid fermentation and extraction from mycelia are practical methods to obtain triterpenoids from authentic *Sanghuang*^15,16^. Ultrasound treatment has a strong mass transfer and a cavitation effect, which facilitate the permeation of the solvent molecules into tissue cells to maximize contact with the solute. Because this can significantly improve the yield of active ingredients, it is widely used in the extraction of bioactive substances. However, there have been few reports on the ultrasonic extraction of triterpenoids from traditional *Sanghuang* mycelia^17,18^, and the best extraction processes of triterpenoids produced from different species may differ. In the following report, the parameters of ultrasonic extraction of triterpenoids for authentic *Sanghuangporus sanghuang* were characterized to obtain the optimal extraction scheme. The antioxidant activities of *Sanghuang* triterpenoids were then determined to provide the basis for the development and utilization of this medicinal fungus.

## Results

### The standard representation

**Figure 1:**
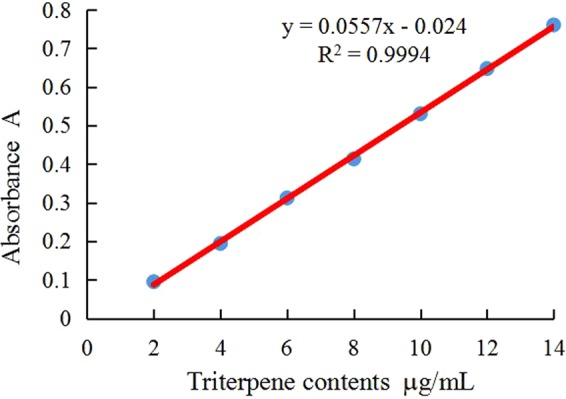
Standard representation of β-amyrin.

The equation of the standard linear function for β-amyrin was y = 0.0557 × −0.02 (Fig. 1), where y is the absorbance value and x is the concentration of triterpenoid sample; the regression coefficient was R^2^ = 0.9994. (The result is shown in Fig. 1).

### Effect of ethanol concentration on triterpenoid extraction

**Figure 2:**
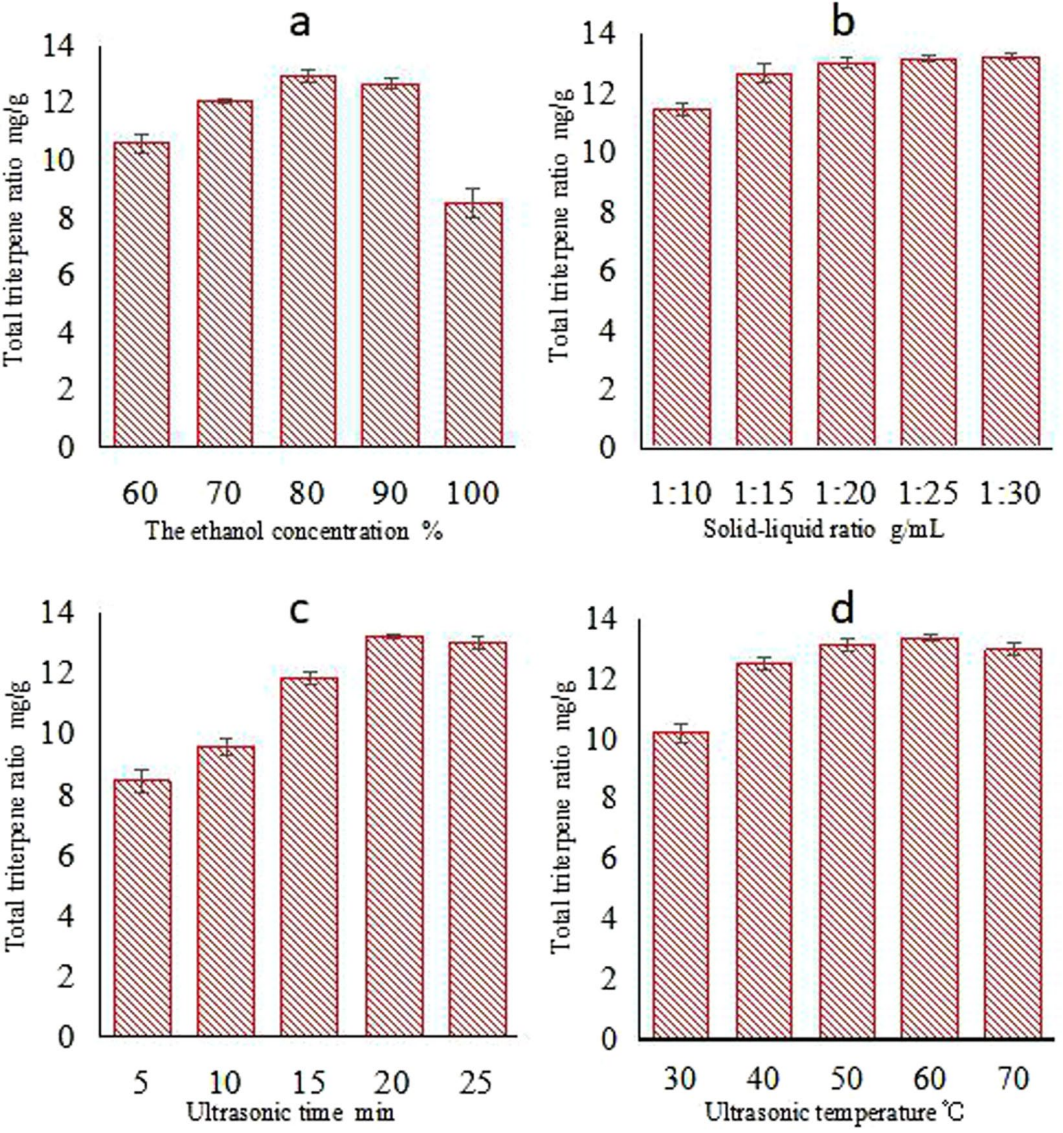
Effect of different factors on total triterpenoid yield from *Sanghuangporus sanghuang*. (**a**) ethanol concentration, (**b**) solid–liquid ratio, (**c**) ultrasonic time, (**d**) ultrasonic temperature.

As the ethanol concentration increased from 60% to 100%, the amount of extracted triterpenoids first increased and then decreased (Fig. 2a). The total triterpenoid extraction was optimal at an ethanol concentration of 80% under the conditions of a 1:15 solid–liquid ratio, 15 min extraction time, 100 W of extraction power, and a 60 °C extraction temperature. When the ethanol concentration was below 60%, water-soluble substances, such as polysaccharides, pigments and pectin, were more dissolved, thus the concentration of triterpenoids was low. Conversely, when the ethanol concentration was higher than 80%, the dissolution of triterpenoids with polar hydroxyl and carboxyl groups was decreased. However, a high concentration of ethanol would increase the cost. Therefore, our results indicated that the best ethanol concentration for extraction was 80%. In this condition, both highly polar water-soluble and lower-polarity ethanol-soluble triterpenoids were extracted together; thus, the highest extraction rate was reached.

### The effect of the solid-liquid ratio on the extraction of triterpenoids

The effect of the solid-liquid ratio on total triterpenoid yield from *Sanghuangporus sanghuang* when using 80% ethanol, a 15-min extraction time, 100 W extraction power, and a 60 °C extraction temperature is shown in Fig. 2b. The total triterpenoid yield markedly increased when the solid-liquid ratio ranged from 1:10 to 1:20. In this condition, the amount of dissolved medicinal powder increased as the solid–liquid ratio increased. When the dissolution of triterpenoid reached an equivalence point, an increase in the solvent volume did not dissolve more triterpenoid; therefore, the extraction yield no longer increased when the solid-liquid ratio reached 1:20. Considering the efficiency and the cost of extraction, the best solid–liquid ratio in this study was 1:20 (g/mL).

### The effect of ultrasound time on total triterpenoid extraction

Ultrasonic waves can help triterpenoids dissolve out of cells. Figure 2c shows that the triterpenoid yield increased significantly as the extraction time increased when a 1:20 (g/mL) solid–liquid ratio, a 60 °C ultrasonic temperature, and 80% ethanol were used. The maximum yield of triterpenoids was obtained when the ultrasonic time was 20 min. After 20 min, most of the cells were dissolved, and the triterpenoid molecules achieved equivalence between the inside and outside of the cells. With extended ultrasonic times, strong mechanical shock can damage the dissolved ingredients, leading to a decrease in the triterpenoid yield. Moreover, longer ultrasonic times can increase the dissolution of alcohol-soluble ingredients, producing a large number of impurities, negatively affecting subsequent purification steps. Therefore, an ultrasonic time of 20 min was used to obtain the maximum extraction yield.

### The effect of ultrasonic temperature on triterpenoid yield

An increase in temperature can enhance the permeability of triterpenoid molecules inside the cell. In the range of 30–60 °C, the total triterpenoid yield increased as the temperature increased when a 1:20 (g/mL) solid–liquid ratio, 80% ethanol concentration, and a 20 min ultrasonic time were used (Fig. 2d). However, the yield slightly decreased when the temperature exceeded 60 °C because, high temperatures (greater than 60 °C) destroy the triterpenoid molecular structure of the five rings. Therefore, an appropriate ultrasonic temperature would maximize the benefits of increasing the temperature, while minimizing the harmful side effects. Thus, 60 °C was used for ultrasonic extraction. (The result is shown in Fig. 2).

### Response surface test design and test results

**Figure 3:**
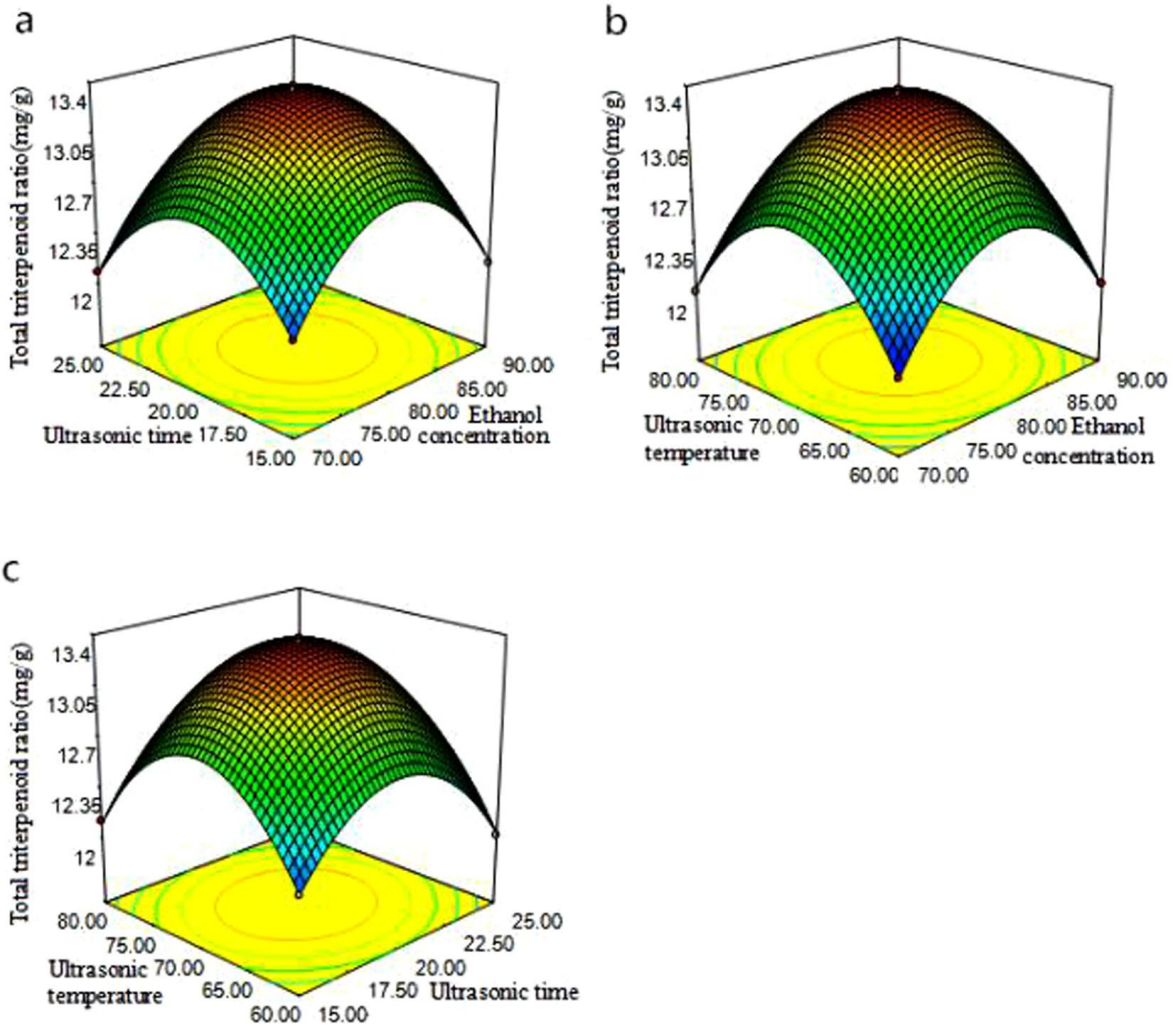
The total triterpenoid ratio from *Sanghuangporus sanghuang* using response surface methodology. (**a**) Ethanol concentrations and ultrasonic times, (**b**) ethanol concentrations and ultrasonic temperatures, (**c**) ultrasonic times and ultrasonic temperatures.

According to the principle of central composite design, the ethanol concentration (X_1_), ultrasonic temperature (X_2_), and ultrasonic time (X_3_) were used as independent variables, and the total triterpenoid yield was used as the response value, with three parameter levels. The extraction process was optimized by using the response surface analysis method. The response surface 3D model was used to analyze the experimental results. The effects of different factors on the total triterpenoid extraction rate, and the degrees of influence of each of the factors are shown in Table 1 and Fig. 3. (The result is shown in Table 1 and Fig. 3).

**Table 1:** Design and response surface methodology results.

Test number	The coding level			Total triterpenoid ratio m g/g
	X_1_	X_2_	X_3_	
1	0	0	0	13.379
2	0	0	0	13.356
3	−1	−1	0	12.183
4	−1	0	1	12.024
5	0	−1	1	12.125
6	0	−1	−1	12.187
7	0	0	0	13.376
8	0	1	−1	12.024
9	1	0	1	12.116
10	−1	0	−1	12.032
11	1	1	0	12.193
12	−1	1	0	12.085
13	1	−1	0	12.152
14	1	0	−1	12.081
15	0	1	1	12.188
16	0	0	0	13.352
17	0	0	0	13.348

The total triterpenoid ratio increased initially, and then declined with an increase in ethanol concentration and ultrasonic time (Fig. 3a). The triterpenoids from *Sanghuangporus sanghuang* have significant polarity, such that the highest extraction efficiency may only be obtained using a solvent with a specific dielectric constant. With an increase in ultrasonic time, the triterpenoid structures were destroyed and the dissolution of other substances was enhanced, which led to disturbances in the triterpenoid extraction efficiency. Figure 3b shows that when the invariant conditions of ultrasonic time, ethanol concentration, and ultrasonic temperature increased, the total triterpenoid ratio first increased and then decreased. Furthermore, the 3D graphical response surface curvature was larger, indicating a more significant effect. As shown in Fig. 3c, when the concentration of ethanol was fixed, the total triterpenoid ratio changed as the ultrasonic time and ultrasonic temperature changed. The 3D curve response surface was larger and the contour line was elliptical, indicating an interaction between the two. The surface effects of ultrasonic temperature, ethanol concentration, and ultrasonic time in the secondary term were significant. The shape of the contour line was elliptical, which indicates the interplay among the many parameters during the total triterpenoid extracting process of *Sanghuangporus sanghuang*, and that these interactions do not involve simple linear relationships.

### Establishment of a model equation and significance test

Design-Expert 7.0.0 software (Stat-Ease, Minneapolis, MN, USA) was used to perform multiple regression analyses of the data. A quadratic equation between the total triterpenoid yield and the ultrasonic extraction variables was obtained as follows: Y = 13.36 + 0.027X_1_ − 0.02X_2_ + 0.016X_3_ + 0.035X1X_2_ − 0.011X_1_X_3_ + 0.057X_2_X_3_ − 0.64X_1_^2^ − 0.57X_2_^2^ − 0.66X_3_^2^. Variance analyses were performed on the regression equation, and the results are shown in Tables 2 and 3. The F-test values were F = 1900.39, (*p* < 0.0001), and the lack of fit F-test was F = 2.36 (*p* = 0.2125). The post hoc test was performed at the single-factor level and a pairwise data analysis was performed. The CV was 0.14%, indicating that the experimental operation was credible; the model determination coefficient R^2^ was 0.9996, indicating a strong correlation between the predicted value and the real value. The adjusted coefficient of determination was 0.9991, indicating that 99.91% of the variability in the data could be explained by the model. The signal-to-noise ratio of the model was 96.559, indicating that the experimental results are highly reliable. Among the three factors, the order terms X_1_, X_2_, and X_3_ were significant. The quadratic terms X_1_^2^ X_2_^2^X_3_^2^ and the cross terms X_1_X_2_, X_2_X_3_, X_1_X_3_ were significant, and the influence degree from strong to weak was ethanol concentration > ultrasound time > ultrasound temperature. These results showed that the linear relationships of the dependent variables and the independent variables were significant and reliable, using the regression equation to describe the relationship between various parameters. (The result is shown in Table 2 and Table 3).

**Table 2:** Variance analysis of regression equations.

Source of variation	Sum of squares	Variance	Mean sum of square	F	P
Model	5.53	9	0.61	1900.39	<0.0001
A-ethanol concentration	5.941E-003	1	5.941E-003	18.37	0.0036
B-ultrasonic time	3.081E-003	1	3.081E-003	9.53	0.0176
C-ultrasonic temperature	2.080E-003	1	2.080E-003	6.43	0.0389
AB	4.830E-003	1	4.830E-003	14.94	0.0062
AC	4.622E-004	1	4.622E-004	1.43	0.2707
BC	0.013	1	0.013	39.50	0.0004
A2	1.72	1	1.72	5307.11	<0.0001
B2	1.37	1	1.37	4240.37	<0.0001
C2	1.84	1	1.84	5683.52	<0.0001
Residual	2.263E-003	7	3.233E-004		
Lack of fit	1.446E-003	3	4.821E-004	2.36	0.2125
Error	8.168E-004	4	2.042E-004		
Total dispersion	5.53	16

**Table 3:** Model fit statistics.

Std. Dev.	0.018	R-Squared	0.9996
Mean	12.48	Adj. R-Squared	0.9991
C.V.%	0.14	Pred. R-Squared	0.9956
PRESS	0.024	Adeq. Precision	96.559

To obtain an extraction equation model of the total amount of triterpenoids in *Sanghuangporus sanghuang*, the first derivative of the regression equation was set to zero; the maximum surface points were used to obtain the optimal extraction conditions of 80.21% ethanol, 19.92 min of ultrasonic treatment, and 60.12 °C ultrasonic temperature to predict a total triterpenoid yield of 13.36 mg/g. To maximize the extraction conditions, they were adjusted to 80% ethanol, 60 °C ultrasonic temperature, and 20 min of ultrasonic time. Three replicates of these extraction conditions were used, with a mean yield of 13.30 mg/g triterpenoids. The experimental values were close to the predicted values, confirming the validity of the proposed model. Our results indicate that the response surface analysis method is suitable for ultrasonic extraction of total triterpenoids from *Sanghuangporus sanghuang*.

### Hydroxyl radical scavenging ability

**Figure 4:**
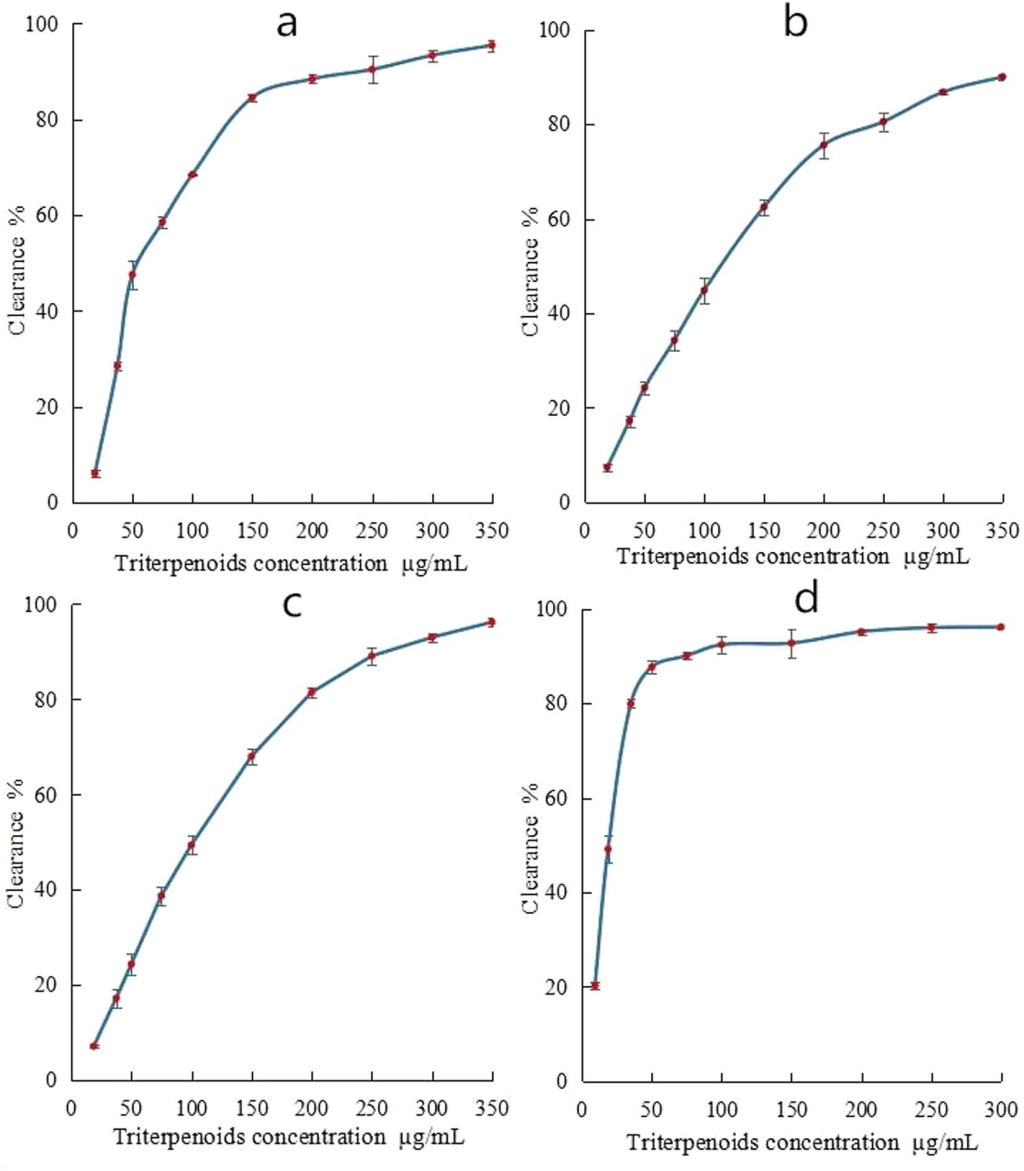
Antioxidative abilities of *Sanghuangporus sanghuang* triterpenoids. (**a**) Hydroxyl radical scavenging ability, (**b**) superoxide anion clearance ability, (**c**) free radical clearance ability of DPPH, (**d**) clearance ability of ABTS.

Reactive oxygen is a general term for a class of chemically-reactive substances. More than 95% of the known free radicals are found in living organisms, including oxygen free radicals such as superoxide anion and hydroxyl radicals, and other molecules such as hydrogen peroxide, singlet oxygen, and triplet oxygen^19^. When the level of reactive oxygen suddenly increases, it can alter the body’s environment and disturb the dynamic balance of macromolecules, causing oxidative stress and damage to lipids, DNA, protein, and polysaccharides, resulting in an increase in toxic substances^20^. Among these toxic substances, hydroxyl radicals are the most reactive oxygen species and one of the most reactive free radicals with respect to the damage of biological molecules^21^. In the present study, we therefore characterized the ability of *Sanghuangporus sanghuang* triterpenoids to scavenge hydroxyl radicals. When the concentration of triterpenoids was 18.75 µg/mL, the clearance rate was only 6%; however, the clearance rate increased to 95% when the concentration of triterpenoids was 350 µg/mL (Fig. 4a), indicating that the hydroxyl free radical scavenging ability increased with triterpenoid concentration.

### Superoxide anion scavenging ability

The superoxide anion has unpaired electrons, which can be produced by *in vitro* autoxidation of pyrogallol. After 30–40 s of oxidation, the concentration of accumulated intermediates is proportional to the reaction time. This linear relationship occurs over 4–5 min. The rate of superoxide anion removal can be characterized by calculating the autoxidation rate, which in turn reflects the antioxidant activity of the natural product^22,23^. Figure 4b shows an increased clearance rate of superoxide anion with increasing concentrations of *Sanghuangporus sanghuang* triterpenoids. The concentration of triterpenoids was 18.75–350 µg/mL, and the corresponding clearance rate increased from about 7% to approximately 90%, indicating that a higher concentration of triterpenoids resulted in a stronger clearing effect.

### Scavenging of DPPH free radicals

2,2-diphenyl-1-picrylhydrazyl (DPPH) is a very stable free radical. An ethanol solution of DPPH is dark purple, and it has a strong absorption maximum at 517 nm. If there is single electron pairing, the absorption disappears, and the degree of fading is quantitatively related to the number of electrons received. Quantitative analysis by spectrophotometry can be used to detect the scavenging of free radicals to evaluate the antioxidant capacity of natural products. In this study, changes in the clearance capacity of DPPH free radicals in *Sanghuangporus sanghuang* liquid cultures were studied. When the triterpenoid concentration was 18.75 µg/mL, the clearance was 6%; when the concentration was 350 µg/mL, the clearance was 96% (Fig. 4c). DPPH free radical scavenging ability gradually stabilized when the concentration of triterpenoid was increased.

### ABTS free radical scavenging ability

2,2′-azino-bis (ABTS) is a water-soluble free radical initiator, which generates stable the blue and green cation-free radical ABTS+ after the oxidation of reactive oxygen, with a maximum absorption peak at 734 nm. When reacted with antioxidants, the ABTS+ will be reduced with decreased color and absorbance of the solution. The lower the absorbance, the stronger the clearance ability of the free radical scavenger, which can be used to evaluate the antioxidant capacity of natural products^24^. The higher the concentration of *Sanghuangporus sanghuang* triterpenoids, the greater the clearance of ABTS. Figure 4d shows that between 9.375–300 µg/mL, the corresponding clearance increased from approximately 20% to 96%. (The result is shown in Fig. 4).

## Discussion

A fermentation production cycle is short, easy to control, produces a large number of cells and metabolites, and has the advantage of large-scale production with automated control without being limited by seasonal climate changes. The present study using a liquid fermentation culture of *Sanghuangporus sanghuang* was able to produce an extensive amount of mycelium in a short time. Ultrasound is a type of elastic mechanical vibration, which has been widely applied in extracting active components from Chinese medicinal materials in recent years. The ultrasonic waves produced by strong vibrations, high acceleration, an intense cavitation effect, and a mixing effect resulted in an increased dissolution of the active ingredients into the solvent. This approach can improve the dissolution rate and shorten the extraction time, to eliminate the necessity of high temperatures during the extraction process, resulting in an optimal extraction methodology. The present study combined single-factor tests and the response surface analysis method for ultrasound-assisted extraction of total triterpenoids from *Sanghuangporus sanghuang*. The extraction was optimized using process conditions including 80% ethanol, an ultrasonic temperature of 60 °C, an ultrasonic time of 20 min, and a solid–liquid ratio of 1:20 (g/mL). Using these conditions, the total triterpenoid yield was 13.30 mg/g. Compared with traditional extraction methods, the extraction time was shorter and the extraction yield of triterpenoids was greater. Zhou (2015) and Jia (2016) used *Ganoderma lucidum* and *Ganoderma applanatum* as raw materials to develop an ultrasonic extraction methodology, with average extraction yields of total triterpenoids of 13.0 and 17.5 mg/g, respectively^25,26^.

The antioxidant activity of triterpenoids is an important index for evaluating their function as medicinal ingredients. In the present study, the antioxidant activity of *Sanghuangporus sanghuang* triterpenoids was evaluated for their ability to remove hydroxyl radicals, superoxide anions, DPPH free radicals, and ABTS free radicals. The hydroxyl radical is a type of free radical with strong reactivity and extreme toxicity. In biological systems, it can react with many substances and damage cells^21^. Superoxide anions can produce free radicals in the body, which can damage cells, leading to the production of hydrogen peroxide and many other reactive substances that can cause serious damage to the body when produced in large amounts^23^. Most exogenous free radicals are reactive and have a very short life span; however, the DPPH free radicals and the ABTS radical are relatively stable properties. The determination of free radical clearance is therefore important for evaluation of the antioxidant activity of *Sanghuangporus sanghuang*^27^. In this study, the free radical clearance increased from 10% to 90% when the *Sanghuangporus sanghuang* triterpenoid concentration was 18.75–350 µg/mL. The ability to resist oxidation was significantly better than that of the *Ganoderma lucidum* triterpenoids. When the concentration of *Ganoderma lucidum* triterpenoids was 0.375–3.000 mg/mL, the oxygen anion clearance increased from 30% to 90%; when the *Ganoderma lucidum* triterpenoid concentration was 0.1875–3.000 mg/mL, the hydroxyl free radical clearance increased from 10% to 90%, and the DPPH free radical clearance increased from 10% to 70%^28^.

The currently used antioxidants are usually synthetic, such as butylhydroxyanisole and 2,6-bis(1,1-dimethylethyl)-4-methylphenol. These antioxidants can improve product stability and maintain product quality, but they also have many disadvantages and may potentially harm humans; thus, identification of novel and safe natural antioxidants is a high priority^5^. The triterpenoids from authentic *Sanghuangporus sanghuang* showed high antioxidant activities and were successfully extracted using ultrasonic waves.

Triterpenoid compounds with a variety of biological activities are important metabolites of *Sanghuangporus sanghuang*, but research on the total triterpenoid substances from authentic *Sanghuangporus sanghuang* is limited. In this study, we measured the optimal fermentation time and ultrasonic extraction process of triterpenoids in laboratory conditions. Future studies should consider different factors, including production costs, production efficiency and stability, fermentation conditions, standardizing extraction technology, and quality testing for industrial mass production. These studies indicated that triterpenoid compounds are predominant constituents responsible for antioxidant activity, but future research should elucidate the bioactive compounds from triterpenoid fractions to establish the biochemical mechanisms and meet large-scale synthetic production. In addition, *in vitro* and *in vivo* drug tests are very different; *in vitro* studies of the antioxidant activity of *Sanghuangporus sanghuang* may show’non-antioxidant’effects in cells and tissues. The antioxidants can prevent or cure a number of pathological conditions. Further *in vivo* experimentation and clinical trials should be undertaken to verify antioxidant activity.

## Conclusion

In our study, a substantial amount of authentic *Sanghuangporus sanghuang* mycelium was obtained in a short amount of time by liquid fermentation. Single-factor tests and the response surface analysis method were used to optimize the ultrasound-assisted extraction process of total *Sanghuangporus sanghuang* triterpenoids, which resulted in a higher extraction yield in a shorter amount of time. The antioxidant activity of *Sanghuangporus sanghuang* triterpenoids indicated that they had a strong ability to remove hydroxyl radicals, superoxide anions, DPPH free radicals, and ABTS free radicals. The results from this study provide a basis for the biological activity characterization of this medicinal fungus, as well as a theoretical basis for its further exploitation and utilization.

## Materials and Methods

### Experimental samples

An authentic strain of *Sanghuangporus sanghuang* (originally isolated from a fruiting body) was obtained from the State Key Laboratory of Mycology (Institute of Microbiology, Chinese Academy of Sciences). *Sanghuangporus sanghuang* hyphae are yellow, its texture is velvety, the reverse is light yellow, and no exudate is produced. The strain was inoculated on a sloping surface of potato dextrose agar (PDA) at 25 °C for 7 days, then the ITS rDNA sequence analysis was verified (GenBank MG209821.1).

### Mycelium fermentation

*Sanghuangporus sanghuang* mycelia were cultivated in a liquid state. The culture medium consisted of 38.96 g/L of corn flour, 25 g/L of glucose, 3.75 g/L of peptone, 4.15 g/L of yeast powder, 20.55 g/L of bran, 1.25 g/L of potassium dihydrogen phosphate, and 0.625 g/L of hydrated magnesium sulfate. The culture medium was divided into 500 mL triangular bottles, with a liquid volume of 250 mL and an inoculation level of 10%. The cultivation used a rotary shaker (28 °C, 150 rpm) in darkness for 10 days. *Sanghuangporus sanghuang* mycelia were dried at 60 °C, and a powder was obtained by grinding and blending.

### Determination of the triterpenoid content

*Sanghuangporus sanghuang* powder (0.1 g) was measured and soaked in an ethanol–water solution. Triterpenoid was extracted by the ultrasonic method (100 W) using different conditions. The ethanol concentrations were 60%, 70%, 80%, 90%, and 100%; the extraction times were 5, 10, 15, 20, and 25 min; the extracting temperatures were 30, 40, 50, 60, and 70 °C; and the solid–liquid ratios were 1:10, 1:15, 1:20, 1:25, and 1:30. The supernatant was collected by centrifuging and the mycelium residue was removed. Then, 0.16 mL of the supernatant was pipetted into a 10-mL centrifuge tube and dried at 70 °C in a water bath. A total of 0.2 mL of newly mixed 5% vanillin–glacial acetic acid solution and 0.8 mL of perchlorate were added and mixed. The solution was heated and reacted in a 70 °C water bath for 20 min, and then rapidly cooled. The solution volume was adjusted to 10 mL with ethyl acetate, and the absorbance at 551 nm was measured using a 752 UV spectrophotometer (China Yangzhou Wandong Medical, Yangzhou City, China).

### Determination of standard curves

β-amyrin (0.02 g) was measured and dissolved in 100 mL of 95% ethanol to obtain a 0.2 mg/mL stock solution. Then 0.0, 0.1, 0.2, 0.3, 0.4, 0.5, 0.4, 0.5, 0.6, or 0.7 mL of stock solution was transferred to 0–7, 10 mL centrifuge tubes. After drying in a 70 °C water bath, 0.2 mL of newly mixed 5% vanillin–glacial acetic acid solution and 0.8 mL perchlorate solution were added and mixed. The resulting solution was heated and incubated in a 70 °C water bath for 20 min, then rapidly cooled. The solution volume was adjusted to 10 mL using ethyl acetate, and the absorbance at 551 nm was determined. A standard curve was then made using Excel software (Microsoft, Redmond, WA, USA).

### Response surface method optimization

According to the principle of central composite design, the ethanol concentration (X_1_), ultrasonic temperature (X_2_), and ultrasonic time (X_3_) were used as independent variables, and the total triterpenoid yield was used as the response value, with three parameter levels. Seventeen sites of response surface analyses were designed (12 of them for the factorial experiment, five for the test center), and the test error was evaluated by central experiment statistics. The X_1_, X_2_, and X_3_ were coded as follows: X_1_ = (X_1_ − 80)/10, X_2_ = (X_2_ − 20)/5, and X_3_ = (X_3_ − 60)/10. The experimental Box–Behnken parameters are shown in Table 4. (The result is shown in Table 4).

**Table 4:** Box–Behnken design of experimental parameter levels.

Parameter	Coding level		
	−1	0	1
Ethanol concentration X1/%	70	80	90
Ultrasonic time X2/min	15	20	25
Ultrasonic temperature X3/°C	50	60	70

### Hydroxyl radical scavenging ability

A hydroxyl free radical kit (A018, produced by the Institute of Biological Engineering in Nanjing, China) was used to determine the hydroxyl radical scavenging ability. The Fenton reaction is a commonly used chemical reaction of hydroxyl free radicals. The amount of hydrogen peroxide is directly proportional to the amount of hydroxyl free radicals generated in this reaction. The generated hydroxyl free radicals react with added or existing electron acceptors. After the reaction, a red material is formed after adding the Griess reagent, and the color intensity is proportional to the concentration of hydroxyl radicals. According to specific steps of the kit instructions, triterpenoid samples were extracted with different concentrations of 18.75, 37.5, 50, 75, 100, 150, 200, 250, 300, and 350 µg/mL. The clearance rate of hydroxyl radicals was obtained using the following formula: clearance rate (%) = (1−absorbance of the triterpenoid samples/absorbance of the control) × 100.

### Determination of the superoxide anion scavenging ability

The determination of the superoxide anion scavenging ability was based on the method described by Li *et al*.^29^. Different concentrations of 0.1 mL triterpenoid samples were pipetted into 1.8 mL of 50.0 mmol/L Tris-HCl buffer (pH 8.2), and then 2.0 mL of deionized water was added to each sample and mixed. After the solution was incubated in a 25 °C water bath for 10 min, 0.1 mL of 10.0 mmol/L pyrogallol solution was added as the chromogenic reagent, and the absorbance at 325 nm was determined immediately. All samples had three replicates, and the mean value of the absorbance was calculated. The absorbance was recorded from 30 s to 4 min, at 30 s intervals, after the addition of pyrogallol, and then plotted as a function of time for the triterpenoid samples to obtain a value for the slope. A similar method was applied for the control group, in which deionized water was used instead of the supernatant as the control solution. According to the following formula, the clearance rate of the superoxide anion was calculated using 18.75, 37.5, 75, 100, 200, 250, 300, or 350 µg/mL of the triterpenoid solution. The clearance rate is as follows: clearance rate (%) = (1−the slope of triterpenoid sample/the slope of the control) × 100.

### Determination of the 2,2-diphenyl-1-picrylhydrazyl (DPPH) free radical scavenging ability

DPPH free radical scavenging ability was determined by the method of Brand–Williams *et al*.^30^. DPPH (20 mg) was measured and dissolved in anhydrous ethanol and adjusted to 250 mL to obtain a solution of 2.0 × 10^−4^ mol/L DPPH. Two milliliters of the supernatant and 2.0 mL of 2.0 × 10^−4^ mol/L DPPH solution were added to the same plug test tube and mixed. The absorbance was measured at 517 nm after a 30 min dark reaction; 2.0 mL of 2.0 × 10^−4^ mol/L DPPH solution and 2.0 mL of absolute ethanol mixture were used as the control groups. The mean value was calculated from three experimental replicates. Based on the following formula, the clearance rate of the DPPH free radical was calculated using concentrations of 18.75, 37.5, 75, 100, 200, 250, 300, or 350 µg/mL of the triterpenoid solution. The clearance rate (%) = (1−absorbance of triterpenoid sample/absorbance of control) × 100.

### 2,2’-Azinobis-(3-ethylbenzthiazoline-6-sulphonate) (ABTS) free radical scavenging ability

The ABTS free radical scavenging ability was determined by the method of Miller *et al*.^31^. An ABTS stock solution of 7.0 mm ABTS and 2.45 mm potassium persulfate solution was prepared. Then, the ABTS working solution was obtained by mixing each of the stock solutions and allowing them to react for 12–16 h at room temperature in the dark. The solution was then diluted by mixing the ABTS radical solution with deionized water to obtain an absorbance of 0.70 ± 0.023 at 734 nm using a spectrophotometer. The solution was also preequilibrated at 30 °C before use. Two mL of sample and 2.0 mL of ABTS working solution were pipetted into a test tube. The mixed solution was reacted at room temperature for 20 min, and the absorbance was measured at 734 nm. Deionized water was used as the control, and the mean value was calculated from three experimental replicates. Based on the following formula, the clearance rate of the ABTS free radical was calculated using 9.375, 18.75, 35, 50, 75, 100, 150, 200, 250, or 300 µg/mL of the triterpenoid solution. The clearance rate (%) = (1−absorbance of the triterpenoid sample/absorbance of the control) × 100.
